# Intraoperative Evaluation of Semiautomatic Localization of the Facial Nerve Using Diffusion Tensor Imaging in Patients with Large Vestibular Schwannomas: A Pilot Study

**DOI:** 10.1055/a-2816-7006

**Published:** 2026-05-08

**Authors:** Wouter D. Maathuis, Tessa M. Kos, Alexander Leemans, Eduard H. Voormolen, Hans G. X. M. Thomeer, Pierre A. J. T. Robe, Tristan P. C. van Doormaal

**Affiliations:** 1 Department of Neurology and Neurosurgery, University Medical Center Utrecht, Utrecht, The Netherlands; 2 Image Sciences Institute, University Medical Center Utrecht, Utrecht, The Netherlands; 3 Department of Otorhinolaryngology, University Medical Center Utrecht, Utrecht, The Netherlands

**Keywords:** DTI, MRI, vestibular schwannomas

## Abstract

**Background:**

Facial nerve damage remains a significant risk during vestibular schwannoma (VS) resection, with reported incidences varying widely (3–46%). Damage risk increases with tumor size. Digital tractography enables nerve reconstruction but typically involves manual procedures, resulting in subjective evaluations that limit reproducibility and validation. We introduce a robust, semi-automatic tractography methodology with reproducible region-of-interest (ROI) generation and present an initial validation using a novel quantitative three-dimensional comparison approach in patients with a large VS.

**Objective:**

To assess the accuracy of facial nerve reconstruction employing a semi-automatic ROI selection method in patients with VSs.

**Materials and Methods:**

We included six patients with an average tumor size of 28 mm (95% CI: 17–40, 100% left) who underwent translabyrinthine VS surgery. Each VS patient was scanned with the regular neuronavigation magnetic resonance imaging (MRI) protocol and a custom diffusion-MRI protocol before surgery. The facial nerve trajectory was reconstructed with a diffusion tensor imaging-based tractography software package using semi-automatic ROI generation. We validated our reconstructions with the Brainlab neuronavigation system for intraoperative point annotation along the course of the facial nerve.

**Results:**

Tracts could be reconstructed in all included patients. The median distance and angle between the points and closest reconstruction were 5.1 mm (IQR: 3.5–7.6) and 38.5 degrees (IQR: 2.7–79.8), respectively.

**Conclusion:**

We present a promising methodology for facial nerve reconstruction in patients with VSs. However, further optimization of the methodology is warranted before a proper clinical validation study can be performed.

## Introduction

Vestibular schwannomas (VSs) are benign cerebellopontine angle tumors originating from the Schwann cells surrounding the vestibulocochlear nerve. Surgical intervention aims at maximizing tumor tissue resection whilst preserving neurological function. One of the main surgical risks is postoperative facial nerve malfunction. The risk of permanent damage ranges from 3 to 46%, depending on tumor size, extent of resection (partial resection vs. gross total resection), and presence of preoperative paresis. 1 2 3

Intraoperative neuromonitoring, consisting of evoked potentials and direct electrical stimulation, is used for the identification of the facial nerve and is the clinical standard to minimize damage. 2 However, literature suggests that the risk of facial nerve damage can be further reduced if the surgeon has a reliable indication of the facial nerve location prior to the surgery. 4

Diffusion tensor imaging (DTI) can be used to reconstruct white matter tracts and cranial nerves using tractography. 5 Multiple studies have investigated the use of DTI-based tractography methods for facial nerve reconstruction in patients with VSs. 6 7 8 9 10 11 12 13 The success rates of DTI-based facial nerve reconstructions are generally high, although the used methods are susceptible to interobserver variability and include subjective outcome metrics. 14 15 16 17 The variability is caused by manual steps in the tractography algorithm, such as region-of-interest (ROI) placement, which is reported to have a large impact on variation in tractography results. 18

In this study, we propose a more robust method for facial nerve reconstruction in patients with VSs. We introduce semi-automatic ROI placement for tractography, based on cloud segmentations, and perform an initial quality assessment of the reconstructions using a novel three-dimensional (3D) validation method.

## Study Design and Population

This prospective study included six patients undergoing surgical resection of a large VS at the department of Neurosurgery in the (BLINDED FOR REVIEW 1), between February and October 2024. The VSs ranged from Koos grade III to IV, 19 and were histopathologically confirmed. The translabyrinthine approach was used during surgery for each patient. Individual patient characteristics and analysis results are provided ( Table 1 ).

**Table 1:** Patient characteristics

Pnt	Sex	Lesion side	Max tumor diameter (mm)	HB-grade before surgery	HB-grade after surgery (+1D)	HB-grade after surgery (latest)	Latest follow-up
P1	F	Left	33	I/II	I	I/II	+12M
P2	F	Left	22	I	I	I	+3M
P3	M	Left	22	I	I	I	+3M
P4	F	Left	30	II	III	I	+3M
P5	F	Left	36	I	III	I	+2M
P6	M	Left	27	I	I	I	+5M

Abbreviations: D, days; F, female; HB, House–Brackmann; M, male; M, months; Pnt, patient.

All patients underwent neuronavigation magnetic resonance imaging (MRI) 1 day before surgery, following standard clinical protocol. 2 Imaging was performed on a 3 Tesla MRI scanner (Ingenia CS, Philips Medical Systems, the Netherlands) and included a T1-weighted gadolinium (T1c) and a custom diffusion-MRI (dMRI) scan. Two different dMRI protocols were used to acquire diffusion data in our patient group. The dMRI sequence acquisition parameters are provided in Table 2 .

**Table 2:** Diffusion scan parameters

Protocol	Patients	Voxel size (mm)	Slice thickness (mm)	#Diffusion gradients	*b* -Value (s/mm ^2^ )
1	1–2	2 × 2 × 2.2	2.2	24 + 1	1,000
2	3–6	2 × 2 × 2	2	32 + 1	1,000

The study complied with the General Data Protection Regulation Act. It was reviewed by the medical ethics board of the (BLINDED FOR REVIEW 1) to determine its applicability under the Medical Research Involving Human Subjects (WMO) Act and was considered as non-WMO applicable research (BLINDED FOR REVIEW 2). Informed consent was provided by all participants prior to their surgical procedure.

### Image Segmentation

Preprocessing of the imaging data, consisting of T1c and dMRI scans, was conducted using 3D Slicer software. 20 The T1c scans were coregistered to the dMRI scan using the Elastix plugin within 3D Slicer. 21 The tumor and cerebellum were automatically segmented from the T1c scans using dedicated software (Lumi, Augmedit, Naarden, the Netherlands). The segmentation algorithms were validated in previous studies. 22 23 In the same image series (T1c), the most medial edge of the ipsilateral porus acousticus was annotated manually by marking its anterior and posterior walls. The maximum tumor diameter in the posterior fossa was manually measured in the transversal plane of the T1c MRI.

### Semi-Automatic Region-of-Interest Generation

**Fig. 1:**
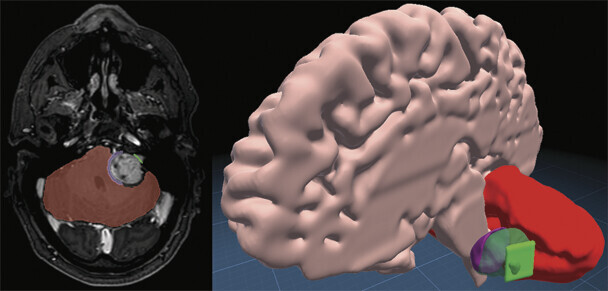
Axial and 3D view of ROIs: porus acousticus (green), tumor (purple), and cerebellum (red). Source: Created by LUMI Prepare (Augmedit, Naarden, the Netherlands).

The segmented structures and porus acousticus markers were converted into three ROIs using a custom MATLAB script (R2024a, Natick, Massachusetts, United States; Fig. 1 ). The first ROI was defined as the most medial third of a shell surrounding the tumor segmentation (purple in Fig. 1 ). The second ROI was a square with sides equivalent to the distance between the two porus acousticus markers (green in Fig. 1 ). The square was oriented such that the markers were centered on opposite sides and it was parallel to the sagittal plane. The third ROI consisted of the entire cerebellum segmentation (red in Fig. 1 ).

### Tractography

Diffusion tensor estimation and fiber tractography were performed using the integrated deterministic tractography algorithm in ExploreDTI. 24 25 The cerebellar ROI was used as an exclusion region, and the other two ROIs as inclusion regions. The fibers were reconstructed using a fractional anisotropy threshold of 0.1, an angle threshold of 40 degrees, and a fiber length range of 30 to 150 mm.

### Validation

Intraoperatively, the facial nerve was identified by the surgeon either visually or using the neuromonitoring probe. The neuronavigation system (Brainlab, Feldkirchen, Germany) was used to mark the course of the facial nerve at three points: the brainstem, the midpoint of the tumor, and at the porus acousticus. Both the point at the brainstem and the midpoint of the tumor were marked during tumor debulking. The point at the porus acousticus was marked after debulking.

**Fig. 2:**
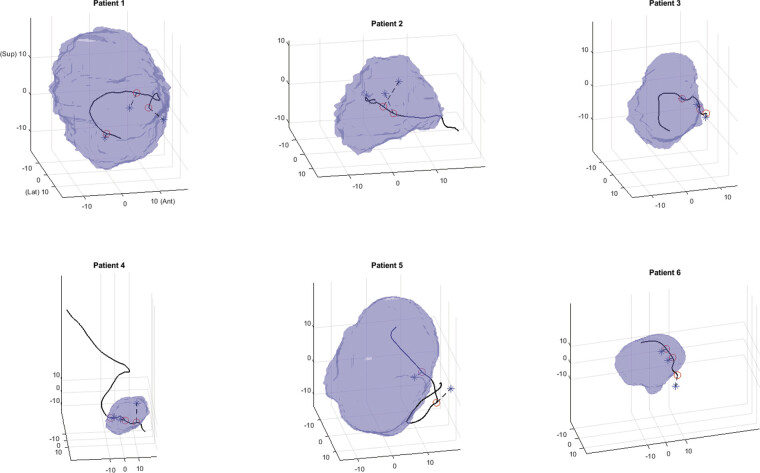
Three-dimensional model of the results in all patients containing the tumor (blue, opaque), closest tract reconstruction (black), intraoperatively annotated points (blue asterisks), and the respective corresponding points on the reconstructed tract (red circles). Each reconstruction is oriented in the same way, where the positive *x* , *y* , and *z* axes are, respectively, anterior (Ant), lateral (Lat), and superior (Sup).

**Fig. 3:**
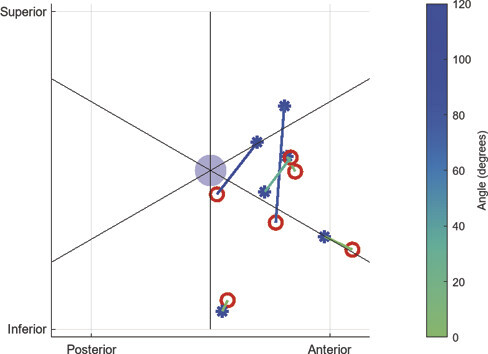
Sagittal view of tumor (light blue), intraoperative control points (blue asterisks), and tract reconstructions (red circles). Corresponding control points and reconstructions are connected with a line. The line is colored based on the angle between the two points with respect to the midpoint of the tumor. Note that the size of the tumor is not to scale.

The annotated points were compared to the closest preoperatively reconstructed fiber tract, and the average distances between two corresponding points were calculated ( Fig. 2 ). We assessed the angle between the control points at the midpoint of the tumor and the corresponding reconstructed tract in the sagittal plane, using the midpoint of the tumor as origin ( Fig. 3 ).

## Results

Tractography reconstruction was successful in all six patients (100%; Fig. 2 ). The reconstruction accurately predicted the nerve to be anterior to the tumor in all patients. A total of 17 control points were intraoperatively marked to delineate the course of the facial nerve ( Fig. 2 ). The median distance between the intraoperative control points and the nearest reconstructed fiber tract was 5.1 mm (IQR: 3.5–7.6). The individual results are summarized in Table 3 .

**Table 3:** Patient characteristics and individual results of the distance between reconstruction and control points

	P1	P2	P3	P4	P5	P6	Median (mm)	IQR1 (mm)	IQR3 (mm)
Max tumor diameter (mm)	33	22	22	30	36	27	–	–	–
Distance at stem (mm)	1.2	10.3	0.3	6.0	4.0	0.9	2.6	1.0	5.5
Distance at midpoint (mm)	4.9	11.2	1.6	5.1	8.9	2.6	5.0	3.2	8.0
Distance at porus (mm)	5.1	6.4	1.3	12.9	NA	6.7	6.4	5.1	6.7
Mean distance (mm)	3.7	9.3	1.1	8.0	6.5	3.4	5.1	3.5	7.6

Abbreviations: IQR, interquartile range; P, patient.

Six control points were placed at the midpoint of the tumor (one in each patient). We found an average angle of 38.5 degrees (IQR: 2.7–79.8) between the control points and reconstructions. ( Fig. 3 )

We observed no apparent differences between the two different dMRI protocols in our patients.

## Discussion

This study investigated DTI-based tractography for reconstruction of the facial nerve in patients who underwent single-sided translabyrinth VS surgery. We introduced novel ROI placement and validation methods, and were able to successfully reconstruct tracts in all patients with a median error of 5.1 mm (IQR: 3.5–7.6). The study cohort consisted of six patients, all of whom had large (>20 mm) tumors located on the left side and classified in Koos Grade III or IV. The facial nerve was consistently identified as anterior to the tumor, aligning with common anatomical presentations. 26

We calculated the angle between the reconstruction and control points. The most relevant evaluation point is at the midpoint of the tumor, which is also where previous studies evaluated the nerve position. 14 17 However, this is also where the effect of tissue shifts due to debulking of the tumor is most prominent, potentially ranging from. This could, combined with neuronavigation system inaccuracies, explain the relatively large average angle of 38.5 degrees (IQR: 2.7–79.8). 27

This study successfully used automated ROI placement, based on automatic segmentations for the definition of the tumor and cerebellar ROI. This approach has the potential to reduce operator dependence and interobserver variability, both of which are reported limitations in the current literature regarding the clinical implementation of tractography for facial nerve visualization. 5 18 However, automatic ROI placement may reduce specificity, leading to an increased number of false-positive tracts. This was also observed in this study, which complicated the interpretation of the reconstruction results and presents a new challenge for clinical implementation.

The morphology of the facial nerve is greatly impacted by the tumor size. Large VSs (>20 mm), as seen in our cohort, compress and flatten the neighboring nerve ( Table 1 ). The surgeon avoids full exposure of the nerve in these cases in order to preserve nerve function. Slightly larger localization errors might be acceptable in these cases since total resection is not the sole goal of surgery in large VSs. 2

The change in morphology also makes tracking with DTI more difficult and influences the accuracy of the intraoperative localization. 8 This partially explains the median distance between corresponding points of 5.1 mm (IQR: 3.5–7.6) measured in our study population. However, the median result is highly influenced by the results in patients 2 and 4, who did not have the largest tumors. We speculate that their metrics were disproportionately impacted by tissue shifts, but point localization inconsistencies by the surgeon and registration inaccuracies in the neuronavigation system could also have impacted these findings. 28

To our knowledge, one other study made use of a neuronavigation system to validate their tractography results. In their study, the facial nerve was manually segmented based on tractography and other imaging. During surgery, they registered a total of 19 control points across five patients and reported a median distance to the reconstructions of 1.21 mm (IQR: 0.81–3.27). 29 However, it is important to note that their tractography algorithm failed in three out of five patients. In all of these patients, the tumors were categorized as “very large.” Although slightly better, the reported distances are based on fewer patients, manual expert intervention in the segmentation, and exclude very large tumors.

### Limitations

This study has several limitations. The main limitation of this study was the number of included patients. Despite the small sample size, the cohort was representative of the broader VS patient population eligible for surgery. 26 However, there was an absence of posteriorly located facial nerves in our patient population. Posterior displacement of the facial nerve in VS is rare, reported in only 0.3 to 3.8% of cases. 30 This rarity underscores the need for larger studies to comprehensively evaluate reconstruction accuracy and to account for such anatomical variations.

We did not explicitly record the fiducial registration error (FRE) or target registration error (TRE) of the neuronavigation system. Literature-based estimates indicate an average FRE and a TRE between 3 and 5 mm, 27 closely matching our observed reconstruction errors. Consequently, achieving substantial further reductions in reconstruction error will require quantifying and addressing inaccuracies inherent to the neuronavigation system itself. 29 The impact of neuronavigation registration error on tract accuracy may be mitigated by intraoperative MRI or setting limits to a maximum FRE.

Neuronavigation-based validation had inherent limitations. The TRE can increase by several millimeters with the duration of surgery, negatively impacting the accuracy of the neuronavigation system. 27 Similarly, tumor debulking causes tissue shifts, which can also impact the accuracy. One review reports tissue shifts ranging from 1 to 20 mm, depending on the type of tumor and measurement location. 28 The marking of our control points was only possible during/after schwannoma debulking due to the anterior displacement of the nerve and translabyrinthine approach. This might explain the localization error found in our study.

Included patients were scanned with two slightly different dMRI protocols ( Table 2 ). No apparent differences were observed between patients scanned with the different protocols. Both protocols closely resembled protocols used in the literature. 7 12 17

### Future Directions

To enhance the clinical utility of facial nerve tractography in VS patients, future work should prioritize minimizing false positives and refining control point registration prior to large-scale validation. Eliminating all false positives without expert input is unlikely; therefore, visual verification by experienced surgeons will remain essential. Nonetheless, reducing the number of candidate tracts—via improved ROI specificity (e.g., automated porus acousticus detection, optimized ROI size) and refined tractography parameters—can streamline this process. Simultaneous intraoperative annotation of all control points could improve consistency, although this may be constrained by tissue shift after tumor debulking.

**Fig. 4:**
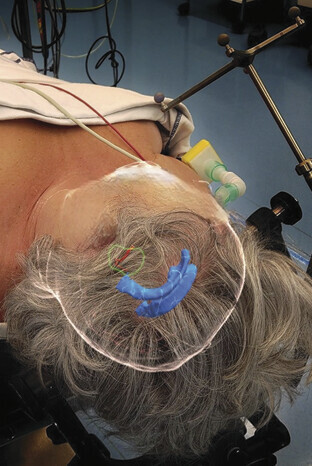
Reconstructed facial nerve (red), schwannoma (green), and ventricles (blue) visualized using AR and overlayed onto a patient.

The software used for segmentation (Lumi) allows for the visualization of the reconstructed tracts in augmented reality. These can be overlayed onto the patient, potentially further improving the spatial interpretation and localization efficiency of the neurosurgeon ( Fig. 4 ).

Facial nerve tractography will serve a complementary role to intraoperative neuromonitoring and neurostimulation. While tractography provides valuable preoperative insight into the expected nerve trajectory, intraoperative techniques remain essential for real-time functional confirmation and nerve preservation.

## Conclusion

The integration of automatic ROI placement and quantitative validation provides a promising methodology for facial nerve reconstruction in patients with VSs. However, further optimization of the methodology is warranted before a large-scale clinical validation study can be performed.
